# Effects of 16 Weeks of Methylphenidate Treatment on Actigraph-Assessed Sleep Measures in Medication-Naive Children With ADHD

**DOI:** 10.3389/fpsyt.2020.00082

**Published:** 2020-02-28

**Authors:** Michelle M. Solleveld, Anouk Schrantee, Hee Kyung Baek, Marco A. Bottelier, Hyke G. H. Tamminga, Cheima Bouziane, Reino Stoffelsen, Paul J. Lucassen, Eus J. W. Van Someren, Roselyne M. Rijsman, Liesbeth Reneman

**Affiliations:** ^1^Department of Radiology and Nuclear Medicine, Amsterdam University Medical Center, University of Amsterdam, Amsterdam, Netherlands; ^2^Brain Plasticity Group, Swammerdam Institute for Life Sciences, University of Amsterdam, Amsterdam, Netherlands; ^3^Department of Child- and Adolescent Psychiatry, Triversum, Alkmaar, Netherlands; ^4^Department of Psychology, University of Amsterdam, Amsterdam, Netherlands; ^5^Academic Center for Child- and Adolescent Psychiatry, De Bascule, Duivendrecht, Netherlands; ^6^Department of Sleep and Cognition, Netherlands Institute for Neuroscience, Amsterdam, Netherlands; ^7^Departments of Integrative Neurophysiology and Psychiatry, Center for Neurogenomics and Cognitive Research (CNCR), VU University and Medical Center, Amsterdam, Netherlands; ^8^Center for Sleep and Wake Disorders, Haaglanden Medical Center, The Hague, Netherlands

**Keywords:** sleep, ADHD, methylphenidate, actigraphy, randomized clinical trial

## Abstract

Methylphenidate (MPH) improves behavioral symptoms of attention-deficit/hyperactivity disorder (ADHD). Its effects on sleep, however, are insufficiently known, as trials with MPH in medication-naive children were so far restricted to relatively short trial durations. Here, we assessed effects of prolonged MPH treatment on sleep in medication-naive boys in a 16-weeks double-blind, placebo controlled, multicenter clinical trial with immediate-release MPH (ePOD-MPH trial, NTR3103). Seventy-five *medication-naive* boys, aged 10–12 years, were screened for eligibility using ADHD DSM-IV criteria. Sleep was assessed using actigraphy, diaries and questionnaires prior to randomization, in week 8, and 1 week after trial end. Fifty boys (mean age 11.4y, SD 0.9) were randomized to MPH or placebo. Linear mixed model analysis demonstrated a significant time-by-treatment interaction effect (p = 0.007) on sleep efficiency. Post-hoc analyses demonstrated that the two groups did not differ from each other (p = 0.94) during treatment (week 8), but that sleep efficiency was significantly improved in the MPH (p = 0.005), but not placebo group (p = 0.87) 1 week after trial end. The lack of MPH's negative effects on sleep during treatment differ from most previous studies and could be explained by the relatively long trial duration in our study and the medication-naive status of our sample; suggesting that evaluating sleep problems only shortly after treatment onset presents an incomplete picture, because it might not be representative for sleep quality after longer treatment periods. Our findings of improved sleep after trial end could be due to rebound effects or longer-term effects of MPH treatment and therefore require replication.

**Clinical Trial Registration:**

Central Committee on Research Involving Human Subjects (an independent registry, identifier NL34509.000.10) before enrollment of the first subject and The Netherlands National Trial Register, identifier NTR3103.

## Introduction

Attention-deficit/hyperactivity disorder (ADHD) is a neurodevelopmental disorder characterized by inattention, hyperactivity, and impulsivity (1). Methylphenidate (MPH) is the most frequently prescribed stimulant medication for ADHD treatment and is very effective in alleviating many ADHD symptoms. MPH blocks the dopamine (DA) transporter, thereby increasing extracellular DA levels, which is thought to underlie the changes in behavioral symptoms (2). Previous studies indicate a five-fold higher prevalence of sleep problems in children with ADHD compared to healthy children (3). These problems include high bedtime resistance, delayed sleep onset, frequent nocturnal awakenings, and excessive daytime sleepiness in ADHD (4–6). However, it is poorly understood whether these sleep problems are part of ADHD symptomatology per se or are induced by ADHD medication, and which neurobiological mechanisms underlie these sleep problems (7).

Although the possibility that sleep problems could be induced by ADHD medication often forms a concern for parents when they discuss the option of pharmacotherapy with the psychiatrist and/or pediatrician (8, 9), studies on the effects of ADHD medication on sleep have so far been inconclusive. Two meta-analyses (10, 11) on objective sleep estimates (including actigraphy and polysomnography) in children with ADHD have concluded that MPH delayed the sleep onset, reduced total sleep time and lowered sleep efficiency relative to placebo-treated patients. However, treatment duration in most earlier studies was relatively short; typically 1–7 weeks, whereas children are generally treated with MPH for months to years. In addition, in most studies children were not medication-naive, which makes it difficult to disentangle effects of ADHD per se from effects of previous medication on these sleep measures.

Neurobiologically, there is considerable support for an effect of MPH on sleep, given the role of the dopaminergic system in sleep-wake regulation. For instance, mice with a deleted dopamine transporter (DAT) gene are more wakeful compared to controls (12), and blocking the DAT with GBR-12909 dose-dependently increases wakefulness (13). This supports that MPH's short term effect is wake-promoting. When considering sleep quality in ADHD, it is important to consider the effects of MPH within the framework of the sleep/wake cycle model of Borbély (1982) and Schwartz and Roth (2008). This model proposes a process S (sleep) and a process C (circadian), that interact to regulate sleep. Process S is defined as sleep pressure (7), which accumulates during the day, and controls the need for sleep after a certain amount of time awake. On the other hand, process C is defined based on a circadian rhythm (14). Previous studies have emphasized that correct dosing as well as the timing of the medication during the day is important to mitigate MPH-related sleep problems (15). MPH administered at the right time and dose might reduce daytime sleepiness often reported in ADHD [as demonstrated in both children and adults (16, 17)] and result in build up of sleep pressure during the day, resulting in improved sleep at night. Unfortunately, most trials conducted so far are relatively short, whereas titration of the correct dose and dissipation of induced sleep problems may take some more time.

Therefore, we here investigated effects of immediate-release MPH on various sleep measures in a cohort of medication-naive ADHD children during a relatively long treatment period. These data were obtained from the “ePOD”-MPH randomized clinical trial (RCT) (Effect of Psychotropic Drugs on the Developing brain) in which medication-naive ADHD patients were randomly assigned to treatment with either MPH or placebo for a period of 16 weeks (18). Actigraphic measures were obtained at week 8 (during treatment) and week 17 (1 week after trial end). In line with previous literature described above, we expected MPH to negatively affect sleep at week 8 of treatment. The expected effect at 17 weeks is more difficult to predict, at least for the MPH group, because medication was stopped and this measurement can have been confounded by rebound effects. Nevertheless, we initially hypothesized that negative effects would be less pronounced or even absent at the end of the trial (week 17) compared to mid-trial (week 8), because one study found that with an increasing duration of the trial, fewer sleep problems were present (11), and because of the hypothesized positive effects of MPH on daytime sleepiness through increased DA turnover. For the placebo group, we expected no changes at week 8 or week 17.

## Materials and Methods

### Trial Design

The ePOD-MPH RCT was a 16-weeks double-blind, randomized, placebo-controlled, multicenter trial with immediate-release MPH treatment in medication-naive children, and adults with ADHD with a blinded end-point evaluation (17). The primary outcome measure of the ePOD-MPH trial was to report on the modification by age of MPH treatment on the outgrowth of the DA system, using state-of-the-art magnetic resonance imaging (MRI) techniques. Our hypothesis was that MPH would induce lasting effects on DA function, one week after treatment end in children, but not in adults, reflecting presumed “neurochemical imprinting” effects, which have been found in rats with the same technique (19). These DA findings have been reported elsewhere (20).

The two secondary outcome measures were: (a) 2 functional outcome measures (fMRI and a neuropsychological test battery (18) and (b) effects of MPH on sleep, which we report here. Because of incomplete sleep data in the adult groups, only the children with complete data sets were included in the current study.

The institutional review board of the Academic Medical Center (AMC) approved the study. The trial ended on June 15, 2015 and was monitored by the Clinical Research Unit of the AMC. Written informed consent was obtained from the legal representatives of all participants.

### Participants

Participants were 50 medication-naive boys (10–12 years old) recruited through clinical programs at the Departments of Child and Adolescent Psychiatry at Triversum (Alkmaar, the Netherlands) and De Bascule Academic Center for Child and Adolescent Psychiatry (Amsterdam, the Netherlands). All participants were diagnosed with ADHD according to DSM-IV (1) by an experienced psychiatrist (MB). The Diagnostic Interview Schedule for Children (authorized Dutch translation)(21) was used to confirm the diagnosis. Subjects with comorbid Axis-I psychiatric disorders requiring medication treatment, a history of major neurological or medical illness, or a history of clinical treatment with drugs influencing the DAergic system (e.g., stimulants, neuroleptics, antipsychotics, and DA receptor type 2 and 3 agonists) were excluded.

### Intervention, Randomization, and Blinding

Participants were randomly assigned (1:1) using a permuted block randomization scheme generated by the local Clinical Research Unit to either immediate-release MPH or matching placebo (PLAC) treatment for 16 weeks. Allocation was concealed for all parties. Placebo and MPH tablets were similar in appearance and were manufactured according to Good Manufacturing Practice criteria. After study end, blinding was checked with the patient and his psychiatrist as well as the study investigators.

The hospital pharmacy (Alkmaar) assigned participants to a specific allocation, using sequentially numbered containers. Participants as well as care providers and research personnel were blinded. The placebo tablet was identical to the MPH tablet with respect to appearance and was manufactured and labeled according to GMP guidelines (2003/94/EG). The treating physician prescribed the medication under double-blind conditions on clinical guidance (reduction in ADHD symptoms), in accordance with Dutch treatment guidelines. That is, children received oral dosages of MPH starting with 0.3 mg/kg day in 1–2 doses and dosages were increased weekly with 5–10 mg/day to a maximum of 40 mg daily until target clinical dosage was reached. If, after in- or decreasing the dosage, serious side effects occur, the patient returned to the previous dosage and dosage modifications will be more gradual thereafter. Decisions about dosage modifications were always and only taken by the treating psychiatrist. Parents of the children received psycho-education. Adherence to the study medication was monitored at 5 control visits (in week 1, 2, 3, 8, and 12).

### Outcomes

Our main outcome measure was estimated sleep efficiency (SE) prior to-, during-, and one week after treatment discontinuation, assessed using actigraphy. SE best summarizes the quality, composition, continuity, and consolidation of sleep (22). Secondary sleep outcome measures were; sleep onset latency (SOL), total sleep time (TST), total in-bed time (TIB), objective and subjective sleep start time (SST and SST-SUBJ), objective and subjective final wake time (Wake time and Wake-subj), wake after sleep onset (WASO), number of wake bouts (WBnumber), mean wake bout time (WBmean), interdaily stability (IS), intradaily variability (IV), the amount of activity during the 5 h with the lowest activity (L5) and during the 10 h with the highest activity (M10), the amplitude of the sleep-wake rhythm (AMP) (**Table 1**).

**Table 1:** Sleep variable definitions.

Variable	Definition
*Actigraph-based sleep estimates*	
Sleep efficiency (SE)	The objective total sleep time divided by the objective time in bed, multiplied by 100 (%)
Sleep onset latency (SOL)	The time it took the subject to fall asleep: time between lights off time (diary) and objective sleep start time (min)
Total sleep time (TST)	The total time period scored as “sleep” between objective sleep start time and objective final wake time (min)
Total in-bed time (TIB)	Time between in bed time (diary) and out of bed time (diary) (min)
Sleep start time (SST)	The objective time when the subject fell asleep (time)
Final wake time (Wake time)	Objective time when the subject woke up in the morning (time)
Wake after sleep onset (WASO)	The total time period scored as “wake” between the objective sleep start time and the objective final wake time (min)
Number of wake bouts (WBnumber)	Number of continuous blocks, one or more “wake” epochs in duration, between the objective sleep start time and the objective final wake time (number)
Mean wake bout time (WBmean)	“Wake after sleep onset (WASO)” divided by the “number of wake bouts” (WBnumber) (min)
*Subjective diary-based sleep estimates*	
Subjective sleep start time (SST-SUBJ)	The subjective time when the subject turned off the lights to go to sleep (time)
Subjective final wake time (Wake-SUBJ)	The subjective time when the subject woke up in the morning (time)
*Actigraphic rest-activity rhythm variables*	
Interdaily stability (IS)	The predictability of the 24-h rest-activity pattern
Intradaily variability (IV)	The fragmentation of the activity profile into brief periods of rest and activity
Activity during 5 h with lowest activity (L5)	The amount of activity in the 5 h with the lowest activity
Onset time of L5 (L5 onset)	The objective time when the 5 h with the lowest amount of activity started
Activity during 10 h with highest activity (M10)	The amount of activity in the 10 h with the highest activity
Onset time of M10 (M10 onset)	The objective time when the 10 h with the highest amount of activity started
Amplitude of the sleep-wake rhythm (AMP)	Amplitude of the sleep-wake rhythm, calculated non-parametrically by subtracting L5 from M10

**Figure 1:**
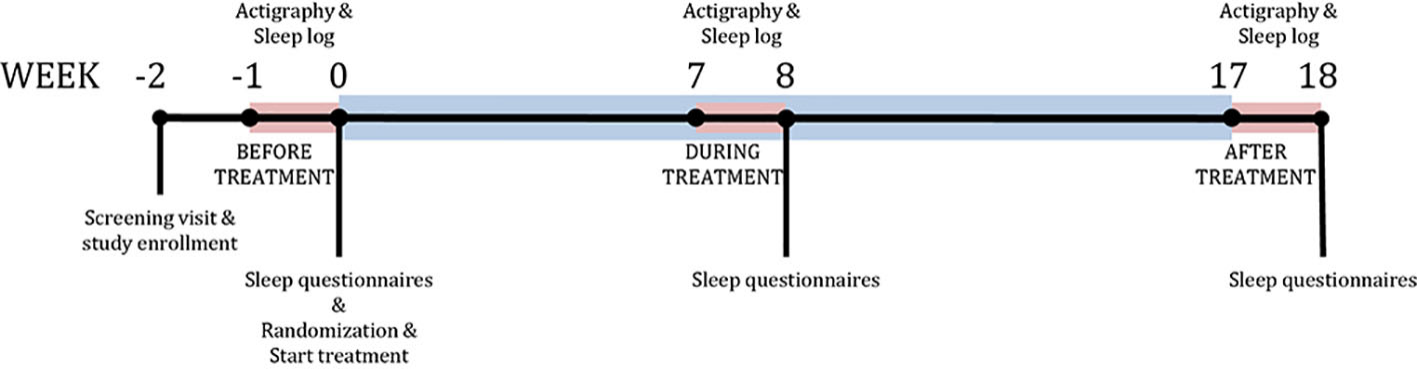
Study design. Timeline of the study. Blue bar represents treatment period. Red bars represent sleep measurement periods using the actigraph.

To obtain these data, subjects wore an Actiwatch AW4 (CamNtech Ltd., Cambridge, UK) on the non-dominant wrist for 24 h/day for five consecutive nights at three time-points during the 16-week treatment: 1 week before randomization (baseline, BL), during the eighth week of treatment (during treatment, DT) and during the week after treatment, i.e., in week 17 (post-treatment, PT) (**Figure 1**). Actiwatch Sleep Analysis 5.0 Software (CNT, UK) was used to calibrate the actiwatches. Epoch length was set to 30 sec. In addition, subjects were instructed to fill in a sleep-diary for five days during each actigraphy period. The actimetry sensor of the ActiWatch measures the gross motor activity of the wrist, with high sensitivity to a palmar-dorsal movement (z-axis). Raw ActiWatch data were extracted and transformed into raw three-dimensional accelerometry data in the unit of counts and were analyzed using MATLAB (23) using the Oakley algorithm (24). In short, a 3–11 Hz band-pass filter was applied prior to sampling at 50 Hz and the data were converted to 128 bins with a resolution of approximately 25 counts/g. Any negative values were set to zero and residual baseline noise was removed. The signal was then converted to unit counts by taking the peak value of each second and summing across the epoch length (i.e., 30 sec). For identifying immobile-mobile (or sleep-wake) pattern, the threshold for MOBILE was set to a medium sensitivity for a window period of 10 min so that the algorithm scores any first 10-min period with more than 1 mobile epoch with activity counts exceeding threshold as MOBILE (or wake). Therefore, the algorithm accommodates only 1 mobile epoch for the given time window during the estimation of sleep onset time and latency. The sleep logs were entered into the program to obtain the subjective sleep parameters for analysis.

In addition to actigraphic sleep estimates, several self-reported questionnaires were administered (**Supplementary Methods and Results**): the Holland Sleep Disorder Questionnaire (HSDQ), used to screen for sleep disorders at baseline; the Epworth Sleepiness Scale (ESS); and the Evaluation List Insomnia Therapy (ELIT) were assessed at all three time points as subjective sleep questionnaire. Severity of restless legs syndrome (RLS), often associated with sleep problems in ADHD, was measured with the Johns Hopkins RLS severity scale (JHRLSS) during the trial. ADHD symptom severity was assessed using the Disruptive Behavior Disorder Rating Scale (DBD-RS).

### Statistical Analysis

A per protocol analysis was conducted, with significance level set at p < 0.05 (2-sided). A linear mixed model [using SPSS 22.0 (IBM, 2013)] was used to estimate the time-by-treatment interaction effect. An autoregressive covariance matrix was assumed, with a fixed intercept and the model was estimated using maximum likelihood. Follow-up pairwise comparisons were corrected for multiple testing using Sidak's correction. As some subjects used melatonin (BL, DT, and PT n = 9) and/or wore the actigraph during holiday periods (BL n = 5, DT n = 10, PT n = 8), these variables were added to the model as dummy-coded covariates. Furthermore, JHRLSS scores were added as covariate. Since there were several missing values for this covariate (18.80% at BL, 22.90% DT and 20.80% PT), we replaced these using logistic regression imputation based on group, age, and JHRLSS score at the two other time-points.

For the sleep variables that were calculated per day (SE, SOL, TST, SST, SST-SUBJ, TIB, Wake time, Wake-SUBJ, WASO, WBnumber, WBmean), days were added as a secondary repeated variable to the model. The ESS and ELIT were not normally distributed and therefore log-transformed. Exploratory analyses were performed to assess possible correlations between the sleep variables and to distinguish between- from within-subject effects (**Supplementary Methods and Results**).

## Results

### Randomisation and Baseline Characteristics

**Figure 2:**
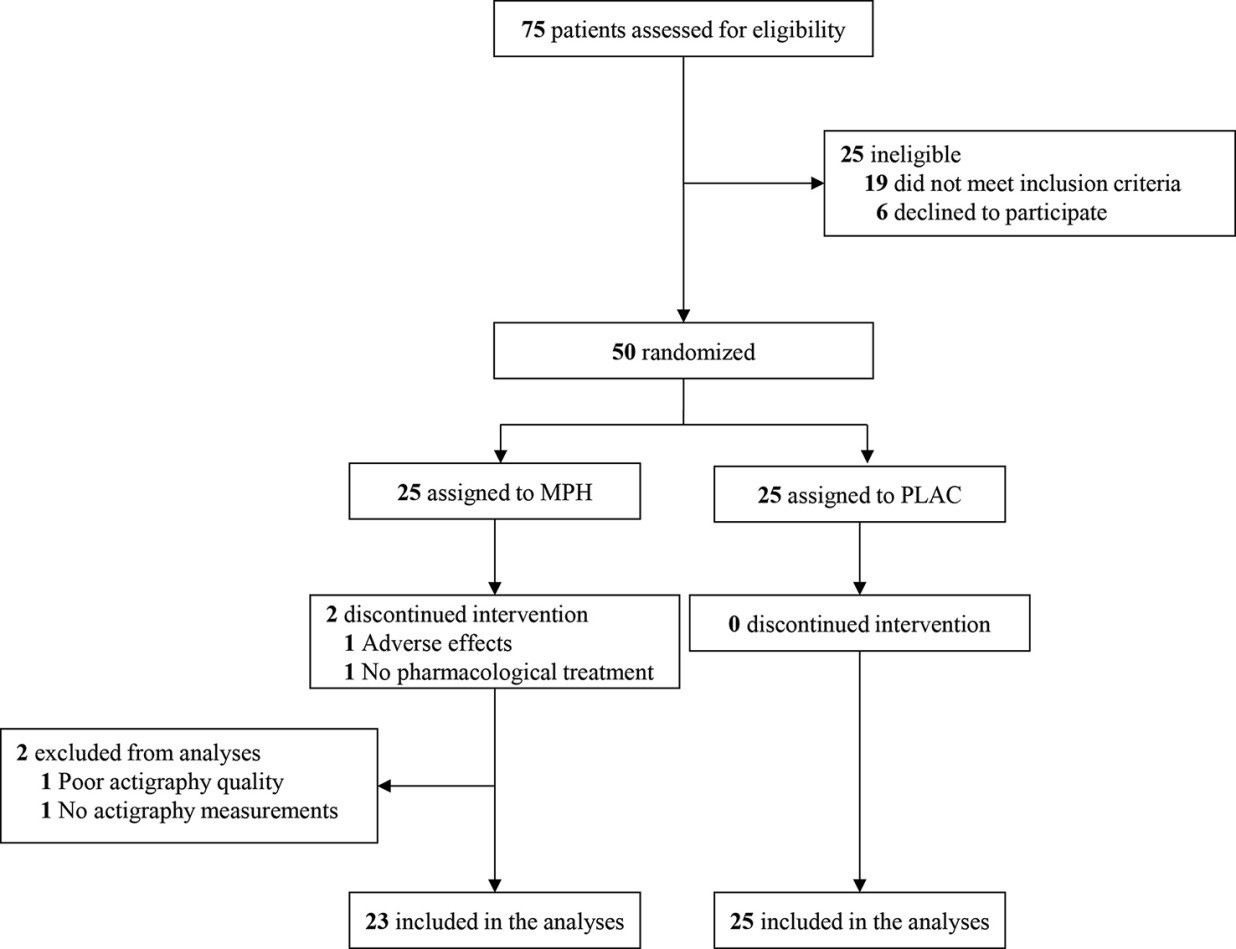
Consolidated standards of reporting trials flow diagram. MPH, methylphenidate condition; PLAC, placebo condition.

Seventy-five children were screened for study inclusion. Fifty subjects met the inclusion criteria and were randomly assigned (1:1) to receive either MPH or placebo treatment (**Figure 2**). Baseline characteristics of the two groups are reported in **Table 2**. DBD-RS scores of attention and hyperactivity did not differ between the two randomisation groups at baseline (attention p = 0.20, hyperactivity p = 0.61). According to the HSDQ, severe sleep problems were absent from our sample at baseline. Adherence to the trial medication did not differ between the two randomisation groups (mean MPH 84.89%, PLAC 79.68%, p = 0.31). The final titrated dose in the placebo group was higher than the one prescribed in the MPH group, but this difference was not statistically different (mean dose MPH 29.13 mg (0.86 mg/kg), mean PLAC 34.40 mg (0.88 mg/kg), p = 0.07).

**Table 2:** Baseline demographics and clinical characteristics of the study subjects.

Variable	Methylphenidate	Placebo
	n = 23	n = 25
*Demographics*		
Age (y), mean (SD)	11.44 (0.80)	11.29 (0.93)
Estimated IQ, mean (SD)	103.22 (21.01)	103.35 (15.05)
ADHD symptom score, mean (SD)		
DBD-RS Attention		
* BL*	21.48 (3.29)	22.72 (3.31)
* DT*	12.48 (5.49)	17.48 (3.08)
* PT*	12.95 (4.85)	17.57 (4.67)
DBD-RS Hyperactivity		
* BL*	15.13 (5.08)	16.00 (6.49)
* DT*	9.42 (4.38)	12.57 (6.40)
* PT*	9.71 (4.44)	12.52 (6.19)
CGI change score, mean (SD)		
* DT*	3.37 (0.83)	3.28 (0.75)
* PT*	3.43 (0.73)	4.00 (0.55)
*Sleep problems*		
HSDQ^A^, No.		
Insomnia	0	1
Hypersomnia	0	0
Parasomnia	0	2
SBD	0	0
CRSD	0	0
RLS	2	3

ADHD, attention-deficit/hyperactivity disorder; CGI, Clinical Global Impression; CRSD, circadian rhythm sleep disorder; DBD-RS, disruptive behavior disorder rating scale; HSDQ, Holland sleep disorder questionnaire; IQ, Intelligence quotient, measured using the Wechsler Intelligence Scale for Children; RLS, Restless legs syndrome; SBD, sleep-related breathing disorder.

^A^5 missing values for HSDQ (MPH n = 4 and placebo n = 1).

### Main Outcome Measure SE

**Figure 3:**
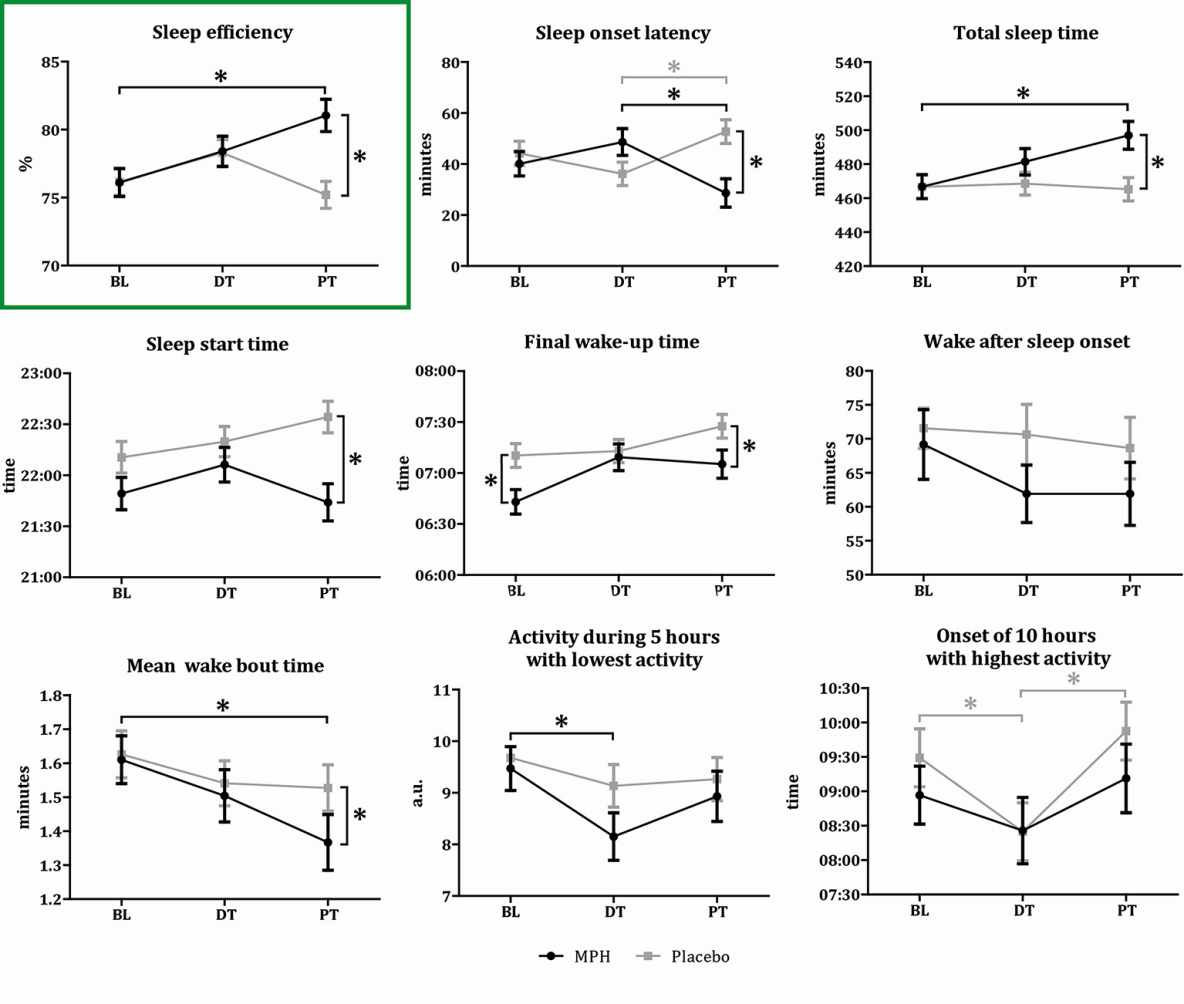
Results sleep variables. Graphical representation of the sleep variable outcomes for the two conditions on three time points (BL, baseline; DT, during treatment; PT, post-treatment). Asterisks indicate a significant difference between the two conditions (p < 0.05). Values represent means per condition for each time point, bars represent the standard deviation. Black lines indicate the MPH treated subjects, gray lines the placebo treated subjects.

Due to logistical problems, sleep data was missing from two participants. Linear mixed model analysis showed a significant time-by-treatment interaction effect on our primary outcome variable SE (F(2,240) = 5.07, p = 0.007). MPH induced an increase in SE at post-treatment when compared to baseline (mean difference 4.94%; 95%CI, 1.18-8.70; p = 0.005). Also, the MPH group had higher SE values than the placebo group (mean difference 5.84%; 95%CI, 2.79-8.88; p < 0.01). At baseline and during treatment (week 8), no differences were found in SE between the MPH and placebo group (BL, mean difference 0.07%, 95%CI -2.88-2.74, p = 0.962; DT, mean difference 0.11%, 95%CI -2.81-3.03, p = 0.94)(**Figure 3** and **Table S1**). Addition of the covariates holiday, melatonin and RLS to the model did not affect our results, although both holiday and melatonin had a main negative effect (holiday F(1,219) = 4.47, p = 0.04, melatonin F(1,145) = 17.68, p < 0.01).

### Secondary Outcome Measures

Significant time-by-treatment interaction effects, or main effects of time or treatment, were found for our secondary outcome variables SOL, TST, SST, Wake time, Wake-SUBJ, M10 onset, and L5. Significance values and post-hoc comparisons for these sleep variables are presented in **Figure 3** and **Tables S1** and **S2**. All these variables demonstrated positive effects in the MPH group at trial end when compared to baseline values as well as to the placebo group. The addition of the covariates did not affect these results, although a time-by-treatment interaction effect for WASO was apparent after addition of the covariates, and not before (**Figure 3** and **Tables S1** and **S2**). For the ELIT sleep subscore as well as for the ELIT wake subscore, we observed a main effect of time, indicating reduced sleep complaints during the trial in both treatment groups. For the ESS questionnaire, we observed a main effect of treatment, which was the result of a decrease in excessive daytime sleepiness induced by MPH when compared to the placebo group (**Table S3** and **Figure S1**).

### ADHD Symptom Severity

We observed a significant time-by-treatment interaction (F(2,87) = 3.33, p = 0.04) on attention symptom severity, indicative of lower ADHD symptoms scores in the MPH group compared to placebo during and after treatment (mean difference DT 4.64, 95%CI 2.14-7.15, p < 0.01, mean difference PT 4.61, 95% CI 2.23-6.98, p < 0.01). Additionally, in both groups, the attention symptom severity was lower during treatment and post-treatment, compared to baseline (DT vs. BL; MPH, mean difference 8.62, 95%CI 6.19–11.06, p < 0.01; PLAC, mean difference 5.29, 95%CI 2.98–7.60, p < 0.01; PT vs. BL; MPH, mean difference 8.5, 95%CI 6.42–10.58, p < 0.01; PLAC, mean difference 5.14, 95%CI 3.15–7.13, p < 0.01). For hyperactivity scores, only a main effect of time was found (F(2,82) = 23.09, p < 0.01), indicating decreased hyperactivity scores in both groups during treatment and post-treatment when compared to baseline (DT vs. BL; mean difference 4.7, 95%CI 2.98–6.43, p < 0.01; PT vs. BL; mean difference 4.62, 95%CI 2.48–6.76, p < 0.01) (see **Table 2**).

## Discussion

In this RCT, we investigated the effects of MPH treatment on various sleep measures in medication-naive boys diagnosed with ADHD. MPH did not affect objective sleep quality mid-treatment (i.e., at week 8). Moreover, children in the MPH group demonstrated a higher sleep efficiency one week after the end of the trial, had a shorter sleep onset latency and slept longer as compared to both the placebo group and baseline. As expected, no changes in sleep efficiency were observed in the placebo group, either at week 8 or week 17. After trial end, participants in both the placebo and MPH groups reported less sleep-related problems. Additionally, MPH treatment improved the attention subscores compared to placebo, whereas hyperactivity improved in both the MPH and placebo group.

The absence of an effect of MPH mid-treatment (week 8) on several sleep variables in boys with ADHD is in contrast with two meta-analyses in children, that found MPH to worsen sleep; which was concluded based on increases in SOL, decreases in SE and an increased number of wake bouts in these studies (10, 11). This discrepancy may be explained by the fact that our first measurement on medication was after 8 weeks, whereas previous studies conducted actigraphy or polosomnography after 1–7 weeks of medication. Interestingly, in line with our observations, one study indeed reported that SE improved when ADHD medication was used for longer periods of time (11). Moreover, the fact that most of these studies had included already medicated children with ADHD may underlie the inconsistency with our current findings. The inclusion of medication-naive subjects in the current study is a critical element in its design, as this excludes any possible interacting effects of prior medication. Using a post-hoc power analysis we found that the effect size of the difference between placebo and MPH at week 8 to have a Cohen's d of 0.01 (resulting in a required sample size >1,000 children per group), suggesting that the likelihood of our finding to be the result of a type-II error to be very small.

One week after trial end, we did observe a positive effect of prolonged MPH treatment on sleep, indicating that the current effects of MPH on sleep outlast the acute effects of the drug. This could result from lasting reductions in ADHD symptoms, e.g., a reduction in attentional problems and thus an altered activity during day time. Indeed, the increased ability to concentrate during the day and a decreased day-time sleepiness may have resulted in an increased build-up of “sleep pressure” (process S), which may have then improved sleep quality at night. Nevertheless, we cannot rule out the possibility that these positive effects result from a “rebound” effect, i.e., a recovery from prior sleep loss. Indeed, an increased sleep propensity (i.e., a shorter sleep onset, longer sleep time) is a hallmark of sleep recovery. However, rebound effects are more likely to occur in the case of sleep deprivation or sleep problems, which were not found in our study for the MPH or placebo group. Unfortunately, in the absence of sleep measures collected in the week prior to trial end we cannot draw firm conclusions based on these findings. Of course, within subject crossover designs would be advantageous in terms of power, but are not ethical with such long treatment durations—therefore most studies using these designs limit the placebo period to 1–2 weeks. The time that children in this study received placebo was therefore dictated by the length of the waiting list (4 months) in the Netherlands at that time (i.e., meaning that children were not withheld effective treatment for longer than in standard clinical care. Notwithstanding, it would be interesting to study children at even longer follow up times whether enduring effects of MPH on sleep are still present after medication is stopped. Preclinical literature on the DA system suggest this might be the case, acting *via* lasting MPH-induced changes in the developing DA system (‘neurochemical imprinting') (19, 25). Consistent with this, we did observe lasting changes in DA function studied before in the same children (20).

The current effects of MPH can be interpreted within the framework of the sleep/wake cycle model of Borbély (14) and Schwartz and Roth (7). Sleep problems in children with ADHD have been previously attributed to changes in either process S (26) or C (27), and increases in SE could be due to changes in either process. However, based on our significant effects of MPH on SE, SOL, TST, SST, and wake time, and due to a lack of effects on the M10 and L5 circadian rhythm sleep variables, it seems more likely that in particular process S is involved in the current effects of MPH on sleep quality in ADHD.

The results of our RCT are of considerable clinical relevance, as possible effects on sleep quality are often an important concern when parents discuss MPH prescription to their children with their psychiatrist or pediatrician (8, 9). Becker et al. (15) reported that children with pre-existing sleep problems can benefit from MPH when titrated at the right dose. Moreover, our results add that the evaluation of sleep problems only shortly after treatment onset might be inappropriate, or at least might not be representative for sleep quality when measured after longer treatment periods (28). Additional studies are needed to further establish the optimal time window for sleep-related effects of MPH, what the effect of drug “holidays” is on sleep, and whether the cessation of medication can change sleep.

There are also some limitations to our study. First, we only included boys of around 11 years old and it thus remains to be demonstrated whether our findings can be generalized to females and other age groups. Although our sample may seem somewhat atypical, it does represent the age group with the highest prevalence of ADHD in boys in the Netherlands. Our sample presented with mild-to-moderate symptoms and therefore the MPH dose prescribed was low-to-moderate. Additionally, based on the HSDQ, no severe (subjective) sleep problems were present in our sample. This could have resulted in more neutral or positive effects of MPH on sleep. It is possible that more severe cases (and those with comorbidities) present at an earlier age and that higher dose regimens result in more negative effects of MPH on sleep. Future studies should investigate whether the current positive effects of MPH on sleep can be extended also to children with ADHD who suffer from more severe ADHD or sleep problems. Moreover, we adminisitered immediate release MPH in this trial and results cannot directly be generalized to extended release medications. For example, Faraone et al. (29) have demonstrated in a recent meta-analysis that the risk for MPH-associated sleep disorders was largest for extended release medications, likely due to pharmacological effects that extend into bedtime. Indeed, using actigraphy it was already demonstrated that triple dosing vs double dosing with MPH decreases total sleep time in children with ADHD, presumably due to a later dosing in time (30); clearly this is a factor that needs to be taken into account both in clinical studies and clinical practice. A second limitation is that some subjects additionally received melatonin during the measurement periods (n = 9 for all three time points). Melatonin is known to positively affect process C (26), and has also been shown to have an inhibitory effect on presynaptic dopamine release (31). Therefore, it was considered a potential confounder and included as a significant covariate in our statistical model for SE, SOL, WASO, WBmean, and IV. This did not change the main results, however, and is therefore unlikely to have influenced our main results. This suggests that the pro-dopaminergic effect of MPH is stronger than the dopamine inhibiting effect of melatonin. Nevertheless, as melatonin use was only present in nine subjects, it is difficult to test its specific effects for this sample and future studies should test interactions between stimulants and melatonin in relation to sleep. Another limitation is that despite our randomization, baseline, and post-treatment wake time differed between the two groups (**Table S1**). Since wake time was significantly later during a holiday than during a school-week (p = 0.009), we evaluated whether treatment groups were differentially measured during holidays. This was however not the case (at baseline: MPH n = 2, placebo n = 3; at follow-up: MPH, n = 4, placebo n = 4) and adding holiday to the model did not alter our findings either.

## Conclusion

In this cohort of medication-naive children with ADHD we show that treatment with MPH does not negatively affect sleep during (week 8) or 1 week after treatment (week 17). Moreover, sixteen weeks of MPH treatment actually increased the efficiency and duration of sleep at 1 week after trial end, but this study could not confirm whether this was a rebound effect or just a long-term effect of treatment. Nevertheless, evaluation of sleep problems associated with ADHD medication should not only be assessed shortly after the onset of treatment, but also after longer treatment periods. Our findings underscore the need for further research, especially in studies with longer MPH treatment durations.

## Author’s Note

The study was carried out at Amsterdam University Medical Centers, University of Amsterdam.

## Data Availability Statement

The datasets generated for this study are available on request to the corresponding author.

## Ethics Statement

The study involving human participants were reviewed and approved by Central Committee on Research Involving Human Subjects The Netherlands. Written informed consent to participate in this study was provided by the participants' legal guardian/next of kin.

## Author Contributions

AS, CB, and HT were responsible for the data collection, with support from MB and RR. HB and MS were responsible for the data processing. MS was responsible for the statistical analyses. AS, CB, HT, ES, LR, MS, and PL interpreted the data. MS, AS, RR, ES, LR, and PL wrote the manuscript. All authors reviewed and approved of the manuscript.

## Funding

This study was funded by faculty resources of the Academic Medical Center, University of Amsterdam, and grant 11.32050.26 from the European Research Area Network Priority Medicines for Children (Sixth Framework Programme). Also the Swammerdam Institute for Life Sciences of the University of Amsterdam supported the study. PJL, AS and LR are also supported by Amsterdam Brain & Cognition.

## Conflict of Interest

The authors declare that the research was conducted in the absence of any commercial or financial relationships that could be construed as a potential conflict of interest.

## Supplementary Material

Figure S1 Results subjective sleep subscores and questionnaire. ESS, Epworth Sleepiness Scale; ELIT, Evaluation List Insomnia Therapy.

## Abbreviations

ADHD, Attention-Deficit/Hyperactivity Disorder; AMC, Academic Medical Center; AMP, amplitude of the sleep-wake rhythm; BL, baseline; DA, dopamine; DBD-RS, Disruptive Behavior Disorder Rating Scale; DT, during treatment; ELIT, Evaluation List Insomnia Therapy; ePOD-MPH, Effect of Psychotropic Drugs on the Developing brain MPH trial; ESS, Epworth Sleepiness Scale; HSDQ, Holland Sleep Disorder Questionnaire; IS, interdaily stability; IV, intradaily variability; JHRLSS, Johns Hopkins RLS severity scale; L5, amount of activity during the 5 h with the lowest activity; MPH, methylphenidate; M10, amount of activity during the 10 h with the highest activity; PLAC, placebo; Process C, process circadian; Process S, process sleep; PT, post-treatment; RCT, randomized controlled trial; RLS, restless legs syndrome; RLS-HTDI, Johns Hopkins telephone diagnostic interview for RLS; SE, sleep efficiency; SOL, sleep onset latency; SST, objective sleep start time; SST-SUBJ, subjective sleep start time; TIB, total in-bed time; TST, total sleep time; Wake time, objective final wake time; Wake-subj, subjective final wake time; WASO, wake after sleep onset; WBmean, mean wake bout time; WBnumber, number of wake bouts; 95%CI, 95% confidence interval.
