# Supplementary material for: Effects of 16 Weeks of Methylphenidate Treatment on Actigraph-Assessed Sleep Measures in Medication-Naive Children With ADHD
Source: Front Psychiatry. 2020 Feb 28;11:82. doi: 10.3389/fpsyt.2020.00082 (PMC7058799; doi:10.3389/fpsyt.2020.00082)
Supplement: Supplementary file 1 [file DataSheet_1.pdf]

Supplementary Material

Randomisation

Randomisation occurred using an in-house developed randomisation program (Clinical Research Unit, Academic Medical Center Amsterdam). Patients were randomly assigned to either treatment (1:1) using a permuted block randomisation scheme. Allocation was concealed for all parties. Placebo and MPH tablets were similar in appearance and were manufactured according to Good Manufacturing Practice criteria. After study end, blinding was checked with the patient and his psychiatrist as well as the study investigators.

Questionnaires

The Holland Sleep Disorder Questionnaire (HSDQ) was used only before start of the treatment as a screening instrument for sleep disorders (insomnia, sleep-related breathing disorder, hypersomnia, circadian rhythm sleep disorder, parasomnia and sleep-related movement disorders) (Kerkhof *et al*, 2013). The Johns Hopkins RLS severity scale was used to measure RLS severity during the trial (Allen and Earley, 2001). The Epworth Sleepiness Scale (ESS) was used at all three time points as an assessment of measuring daytime sleepiness (Johns, 1991). The ESS is a self-administered questionnaire with 8 items on a 4-point scale (0-3). A score of 11 or higher represents increasing levels of excessive daytime sleepiness. The Evaluation List Insomnia Therapy (ELIT) was conducted at all three time points to assess mood changes, and sleep and wake complaints (Kerkhof, 1999). The ELIT is also a self-administered questionnaire with 19 items on a 5-point scale (0-4). Three factor scores can be calculated: mood loss, sleep complaints and wake complaints. The Disruptive Behavior Disorder Rating Scale (DBD-RS) was used to assess disruptive behavior disorders (Pelham *et al*, 1992).

Exploratory correlations

Exploratory correlation analyses for several of the sleep variables that were calculated for each day separate were performed in order to distinguish between within and between subject effects (van de Pol and Wright, 2009). On all time points, a negative correlation was found between SE and SOL for within subject effects, indicating that subjects with mean lower SOL also show a mean higher SE (baseline β=-0.461, p<0.001, during treatment β=-0.540, p=<0.001, post treatment β=-0.615, p<0.001). In addition, between subjects effects on SOL were also negatively correlated to SE, showing that a lower

SOL itself also correlates with a higher SE (baseline β=-0.486, p=<0.001, during treatment β=-0.416, p<0.001, post treatment β=-0.472, p<0.001). Addition of group and holiday to this model did not affect these results. Second, a positive correlation was found on both the within and between subjects effects of TST on SE on all time points (Within subject effects: baseline β=0.542, p<0.001, during treatment β=0.570, p<0.001, post treatment β=0.642, p<0.001. Between subject effects: baseline β=0.386, p<0.001, during treatment β=0.343, p<0.001, post treatment β=0.462, p<0.001). A mean increase in TST correlates with a mean increase in SE (between subjects effect), and an increase in TST correlates with an increase in SE (within subject effect). Addition of group and holiday did not affect these results. Third, on baseline and after treatment, an increase in SST was associated with an increase in SE (within subjects effect, baseline β=0.224, p=0.002, post treatment β=0.245, p=0.001). Addition of group and holiday did not affect these results. Lastly, on all time points, an increase in final wake time correlates with an increase in SE (within subjects effect, baseline β=0.316, p<0.001, during treatment β=0.347, p<0.001, post treatment β=0.338, p<0.001). Addition of group and holiday did not affect these results.

References

Allen RP, Earley CJ (2001). Validation of the Johns Hopkins restless legs severity scale. *Sleep Med* **2**: 239–242.

Johns MW (1991). A new method for measuring daytime sleepiness: the Epworth sleepiness scale. *Sleep* **14**: 540–5.

Kerkhof GA (Den Haag, The Netherlands, 1999). *Evaluatie Lijst Insomnie Therapie*. .

Kerkhof GA, Geuke MEH, Brouwer A, Rijsman RM, Schimsheimer RJ, Kasteel V Van (2013). Holland Sleep Disorders Questionnaire: A new sleep disorders questionnaire based on the International Classification of Sleep Disorders-2. *J Sleep Res* **22**: 104–107.

Pelham WE, Gnagy EM, Greenslade KE, Milich R (1992). Teacher ratings of DSM-III-R symptoms for the disruptive behavior disorders. *J Am Acad Child Adolesc Psychiatry* **31**: 210–8.

Pol M van de, Wright J (2009). A simple method for distinguishing within- versus between-subject effects using mixed models. *Anim Behav* **77**: 753–758.
