# Supplementary figures and images for: Effects of 16 Weeks of Methylphenidate Treatment on Actigraph-Assessed Sleep Measures in Medication-Naive Children With ADHD
Source: Front Psychiatry. 2020 Feb 28;11:82. doi: 10.3389/fpsyt.2020.00082 (PMC7058799; doi:10.3389/fpsyt.2020.00082)

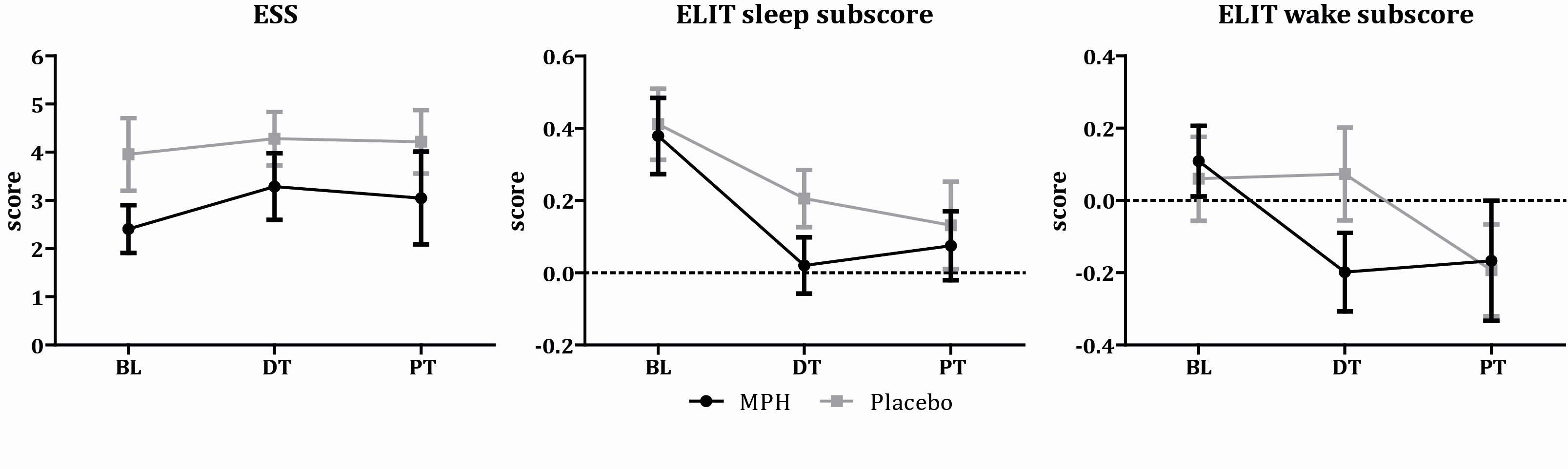

Supplement: Figure S1 — Results subjective sleep subscores and questionnaire. ESS, Epworth Sleepiness Scale; ELIT, Evaluation List Insomnia Therapy. [file Image_1.tiff]
