# Supplementary material for: Effects of 16 Weeks of Methylphenidate Treatment on Actigraph-Assessed Sleep Measures in Medication-Naive Children With ADHD
Source: Front Psychiatry. 2020 Feb 28;11:82. doi: 10.3389/fpsyt.2020.00082 (PMC7058799; doi:10.3389/fpsyt.2020.00082)
Supplement: Supplementary file 4 [file Table_1.doc]

**Table S1. Mean and standard deviations per group per time-point for all sleep variables.**

|  | **BL** | | **DT** | | **PT** | |
| --- | --- | --- | --- | --- | --- | --- |
| **Variable** | **MPH** | **PLAC** | **MPH** | **PLAC** | **MPH** | **PLAC** |
| SE (SD), % | 76.33 (8.38) | 76.15 (6.28) | 78.49 (7.80) | 78.59 (7.23) | 80.59 (7.55) | 75.17 (10.48) |
| SOL (SD), min | 39.71 (41.14) | 42.83 (35.02) | 48.34 (36.10) | 35.88 (29.78) | 31.98 (34.79) | 52.14 (52.95) |
| TST (SD), min | 467.52 (59.73) | 466.68 (53.88) | 481.33 (50.02) | 470.06 (54.11) | 495.91 (56.34) | 465.09 (77.30) |
| TIB (SD), min | 612.63 (55.78) | 613.67 (57.80) | 614.43 (42.26) | 599.08 (59.99) | 616.89 (49.32) | 618.96 (61.64) |
| SST (SD), time | 21:03 (3:45) | 20:47 (5:02) | 21:48 (2:35) | 21:48 (3:04) | 21:27 (2:37) | 19:44 (6:33) |
| Wake-time (SD), time | 6:43 (0:50) | 7:10 (0:49) | 7:09 (0:50) | 7:13 (0:48) | 7:03 (0:42) | 7:26 (1:14) |
| WASO (SD), min | 68.99 (27.22) | 71.78 (19.81) | 62.27 (21.69) | 68.39 (27.69) | 61.41 (26.16) | 69.30 (26.73) |
| WBnumber (SD), # | 43.66 (12.11) | 46.19 (11.73) | 41.94 (12.78) | 44.28 (15.94) | 44.10 (12.80) | 44.76 (14.37) |
| WBmean (SD), min | 1.59 (0.51) | 1.60 (0.45) | 1.50 (0.51) | 1.52 (0.56) | 1.39 (0.50) | 1.56 (0.55) |
| SST-SUBJ (SD), time | 21:03 (0:44) | 20:32 (4:05) | 21:17 (0:42) | 21:25 (2:16) | 21:15 (0:51) | 20:02 (4:58) |
| Wake-SUBJ (SD), time | 7:08 (0:53) | 7:35 (0:48) | 7:23 (0:49) | 7:33 (0:47) | 7:21 (0:42) | 7:54 (1:10) |
| IS (SD) | 0.75 (0.15) | 0.74 (0.16) | 0.76 (0.11) | 0.76 (0.13) | 0.76 (0.16) | 0.73 (0.15) |
| IV (SD) | 0.38 (0.07) | 0.36 (0.09) | 0.40 (0.09) | 0.37 (0.06) | 0.38 (0.08) | 0.35 (0.06) |
| L5 (SD), # | 9.48 (2.04) | 9.55 (1.90) | 8.21 (1.54) | 9.11 (2.20) | 8.92 (2.30) | 9.38 (1.79) |
| L5 onset (SD), time | 14:18 (10:40) | 17:38 (9:21) | 17:26 (9:43) | 16:26 (10:01) | 18:31 (8:40) | 16:43 (9:44) |
| M10 (SD), # | 53.76 (4.43) | 53.25 (5.62) | 54.33 (4.06) | 54.44 (4.48) | 53.37 (5.65) | 53.96 (4.89) |
| M10 onset (SD), time | 8:56 (1:37) | 9:29 (2:05) | 8:25 (1:25) | 8:25 (1:36) | 9:11 (2:43) | 9:52 (2:15) |
| AMP (SD) | 44.29 (4.71) | 43.70 (5.85) | 46.11 (4.18) | 45.32 (4.37) | 44.46 (5.09) | 44.58 (4.64) |
| ELIT mood (SD), score | 1.49 (0.30) | 1.40 (0.40) | 1.35 (0.29) | 1.31 (0.35) | 1.37 (0.41) | 1.26 (0.26) |
| ELIT sleep (SD), score | 1.17 (0.54) | 1.13 (0.53) | 0.72 (0.35) | 0.95 (0.41) | 0.81 (0.44) | 0.85 (0.62) |
| ELIT wake (SD), score | 0.53 (0.47) | 0.40 (0.60) | 0.17 (0.52) | 0.42 (0.68) | 0.20 (0.79) | 0.13 (0.65) |
| ESS (SD), score | 2.61 (2.48) | 3.75 (3.69) | 2.79 (2.90) | 4.30 (2.78) | 3.06 (4.44) | 4.10 (3.31) |
| JHRLSS (SD), score | 0.68 (1.095) | 0.49 (0.972) | 0.70 (1.066) | 0.60 (1.043) | 0.68 (1.053) | 0.58 (1.015) |

Abbreviations: AMP, amplitude of the sleep-wake rhythm; BL, baseline;; DT, during treatment; ELIT, evaluation list insomnia therapy; ESS, Epworth sleepiness scale; IS, interdaily stability; IV, intradaily variability; JHRLSS, Johns Hopkins restless leg syndrome severity scale; L5, activity during 5 hours with lowest activity; L5 onset, onset time of L5; M10, activity during 10 hours with highest activity; M10 onset, onset time of M10; PT, post-treatment; SD, standard deviation; SE, sleep efficiency; SOL, sleep onset latency; SST, sleep start time; SST-SUBJ, subjective sleep start time; TIB, total in-bed time; TST, total sleep time; Wake-SUBJ, subjective final wake time; Wake-time, final wake time; WASO, wake after sleep onset; WBmean, mean wake bout time; WBnumber, number of wake bouts
