# Supplementary material for: Effects of 16 Weeks of Methylphenidate Treatment on Actigraph-Assessed Sleep Measures in Medication-Naive Children With ADHD
Source: Front Psychiatry. 2020 Feb 28;11:82. doi: 10.3389/fpsyt.2020.00082 (PMC7058799; doi:10.3389/fpsyt.2020.00082)
Supplement: Supplementary file 5 [file Table_2.doc]

**Table S2. Linear mixed model results for the secondary sleep variables (fixed effects)**

|  | **Interaction effect** | **Main effects** |  |  |  |  | **Significant post-hoc effects^A^** |
| --- | --- | --- | --- | --- | --- | --- | --- |
| **Variable** | **Time * Treatment** | **Treatment** | **Time** | **Holiday** | **RLS** | **Melatonin** |  |
| SOL | F(2,228)=3.02 ***P=0.003*** |  |  |  |  |  | PT: MPH > PLAC; MPH: PT < DT; PLAC: PT > DT |
| SOL + covariates | F(2,224)=7.05 ***P=0.001*** |  |  | F(1,207)=1.62 P=0.204 | F(1,174)=0.63 P=0.430 | F(1,133)=9.13 ***P=0.003*** | PT: MPH < PLAC; MPH: PT < DT; PLAC: PT > DT |
| TST | F(2,237)=2.41 P=0.092 | F(1,157)=5.29 ***P=0.023*** | F(2,237)=1.57 P=0.210 |  |  |  | PT: MPH > PLAC; MPH: PT > BL |
| TST + covariates | F(2,235)=2.63 P=0.074 | F(1,161)=5.76 ***P=0.018*** | F(2,235)=1.94 P=0.174 | F(1,222)=0.47 P=0.493 | F(1,192)=4.44 ***P=0.036*** | F(1,157)=3.59 P=0.060 | PT: MPH > PLAC; MPH: PT > BL |
| TIB | F(2,254)=1.27 P=0.282 | F(1,128)=0.38 P=0.541 | F(2,255)=1.46 P=0.234 |  |  |  |  |
| TIB + covariates | F(2,252)=1.65 P=0.194 | F(1,131)=0.15 P=0.697 | F(2,253)=1.61 P=0.202 | F(1,232)=1.24 P=0.266 | F(1,187)=0.84 P=0.362 | F(1,128)=3.81 P=0.053 |  |
| WASO | F(2,288)=0.13 P=0.882 | F(1,114)=2.76 P=0.099 | F(2,291)=2.22 P=0.110 |  |  |  |  |
| WASO + covariates | F(2,278)=0.06 P=0.940 | F(1,116)=404 ***P=0.047*** | F(2,281)=2.95 P=0.053 | F(1,257)=1.16 P=0.283 | F(1,195)=0.18  P=0.676 | F(1,114)=7.38 ***P=0.008*** |  |
| SST | F(2,272)=2.15 P=0.118 | F(1,104)=10.07 ***P=0.002*** | F(2,275)=1.13 P=0.324 |  |  |  | PT: MPH < PLAC; PLAC: PT < DT |
| SST + covariates | F(2,257)=3.12 ***P=0.046*** |  |  | F(1,236)=28.69 ***P<0.001*** | F(1,177)=1.06 P=0.304 | F(1,104)=0.23 P=0.635 | PT: MPH < PLAC; PLAC: PT < DT |
| SST-SUBJ | F(2,240)=1.31  P=0.271 | F(1,128)=1.99  P=0.160 | F(2,242)=2.25  P=0.107 |  |  |  |  |
| SST-SUBJ + covariates | F(2,240)=1.25  P=0.289 | F(1,129)=1.61  P=0.207 | F(2,241)=2.17  P=0.117 | F(1,221)=0.75  P=0.386 | F(1,180)=1.39  P=0.239 | F(1,128)=0.63  P=0.428 |  |
| Wake time | F(2,261)=1.69 P=0.186 | F(1,133)=8.02 ***P=0.005*** | F(2,263)=3.91 ***P=0.021*** |  |  |  | BL: MPH < PLAC; PT: MPH < PLAC; MPH: DT > BL |
| Wake time + covariates | F(2,250)=2.82 P=0.061 | F(1,141)=6.33 ***P=0.013*** | F(2,252)=3.21 ***P=0.042*** | F(1,233)=46.11 ***P<0.001*** | F(1,190)=1.02 P=0.315 | F(1,137)=1.24 P=0.268 | BL: MPH < PLAC; PT: MPH < PLAC; PLAC: PT > DT |

**Supplementary Table 2 (continued). Linear mixed model results for the secondary sleep variables (fixed effects)**

|  | **Interaction effect** | **Main effects** |  |  |  |  | **Significant post-hoc effects^A^** |
| --- | --- | --- | --- | --- | --- | --- | --- |
| **Variable** | **Time * Treatment** | **Treatment** | **Time** | **Holiday** | **RLS** | **Melatonin** |  |
| Wake-SUBJ | F(2,273)=1,48  P=0.230 | F(1,119)=11.76  ***P=0.001*** | F(2,275)=2.46  P=0.087 |  |  |  | BL: MPH < PLAC; PT: MPH < PLAC |
| Wake-SUBJ + covariates | F(2,256)=2.68  P=0.070 | F(1,124)=10.57  ***P=0.001*** | F(2,259)=3.01  P=0.051 | F(1,237)=45.46  ***P<0.001*** | F(1,186)=0.61  P=0.437 | F(1,121)=0.29  P=0.590 | BL: MPH < PLAC; PT: MPH < PLAC; PLAC: PT > DT |
| WBnumber | F(2,284)=0.73 P=0.483 | F(1,117)=1.08 P=0.302 | F(2,286)=0.82 P=0.443 |  |  |  |  |
| WBnumber + covariates | F(2,282)=0.75 P=0.472 | F(1,118)=1.01 P=0.317 | F(2,284)=0.80 P=0.450 | F(1,261)=0.01 P=0.929 | F(1,198)=1.29 P=0.257 | F(1,115)=0.38 P=0.541 |  |
| WBmean | F(2,263)=0.58  P=0.559 | F(1,122)=1.12  P=0.291 | F(1,265)=2.64  P=0.073 |  |  |  |  |
| WBmean + covariates | F(2,246)=0.93 P=0.396 | F(1,127)=2.23 P=0.138 | F(2,248)=3.69 ***P=0.026*** | F(1,227)=1.96 P=0.163 | F(1,182)=0.89 P=0.347 | F(1,124)=15.4  ***P<0.001*** | PT: MPH < PLAC; MPH: PT < BL |
| IV | F(2,82)=0.14 P=0.866 | F(1,56)=3.85 P=0.055 | F(1,81)=0.38 P=0.384 |  |  |  |  |
| IV + covariates | F(2,84)=0.32 P=0.728 | F(1,59)=3.90 P=0.053 | F(2,84)=0.44 P=0.647 | F(1,113)=12.13 ***P=0.001*** | F(1,114)=2.45 P=0.120 | F(1,54)=9.29 ***P=0.004*** |  |
| AMP | F(2,91)=0.07 P=0.935 | F(1,59)=0.26 P=0.612 | F(2,91)=1.21 P=0.303 |  |  |  |  |
| AMP + covariates | F(2,90)=0.06 P=0.941 | F(1,59)=0.05 P=0.823 | F(2,90)=2.20 P=0.116 | F(1,111)=10.76 ***P=0.001*** | F(1,98)=0.90 P=0.345 | F(1,54)=1.05 P=0.310 |  |
| IS | F(2,85)=0.10 P=0.905 | F(1,55)=0.11 P=0.746 | F(2,84)=0.29 P=0.752 |  |  |  |  |
| IS + covariates | F(2,85)=0.22 P=0.801 | F(1,55)=0.01 P=0.930 | F(2,85)=0.55 P=0.582 | F(1,114)=6.38 ***P=0.013*** | F(1,102)=2.44 P=0.122 | F(1,49)=1.21 P=0.277 |  |
| M10 | F(2,87)=0.13 P=0.879 | F(1,56)=0.001 P=0.971 | F(2,87)=0.36 P=0.697 |  |  |  |  |
| M10 + covariates | F(2,84)=0.17 P=0.844 | F(1,55)=0.14 P=0.706 | F(2,84)=0.80 P=0.455 | F(1,114)=7.63 ***P=0.007*** | F(1,104)=1.34 P=0.250 | F(1,50)=0.12 P=0.731 |  |
| M10 onset | F(2,83)=0.33 P=0.718 | F(1,49)=1.28 P=0.264 | F(2,81)=3.50 ***P=0.035*** |  |  |  |  |
| M10 onset + covariates | F(2,79)=0.53 P=0.593 | F(1,52)=0.49 P=0.486 | F(2,78)=6.21 ***P=0.003*** | F(1,113)=15.90 ***P<0.001*** | F(1,113)=0.05 P=0.829 | F(1,48)=0.62 P=0.435 | PLAC: BL > DT; PLAC: PT > DT |
| L5 | F(2,71)=0.99 P=0.377 | F(1,48)=0.81 P=0.373 | F(2,70)=4.13 ***P=0.020*** |  |  |  | MPH: BL > DT |
| L5 + covariates | F(2,70)=0.86 P=0.430 | F(1,49)=0.98 P=0.327 | F(2,71)=4.47 ***P=0.015*** | F(1,87)=0.53 P=0.469 | F(1,99)=0.004 P=0.951 | F(1,46)=1.30 P=0.261 | MPH: BL > DT |
| L5 onset | F(2,88)=0.81 P=0.449 | F(1,58)=0.04 P=0.837 | F(2,86)=0.25 P=0.779 |  |  |  |  |
| L5 onset + covariates | F(2,87)=0.78 P=0.461 | F(1,60)=0.12 P=0.730 | F(2,87)=0.31 P=0.733 | F(1,115)=1.36 P=0.245 | F(1,109)=0.05 P=0.828 | F(1,55)=0.08 P=0.775 |  |

Abbreviations: BL, baseline; DT, during treatment; MPH, methylphenidate; PLAC, placebo; PT, post-treatment

^A^ Sidak post-hoc test P<0.05
