# Supplementary material for: Effects of 16 Weeks of Methylphenidate Treatment on Actigraph-Assessed Sleep Measures in Medication-Naive Children With ADHD
Source: Front Psychiatry. 2020 Feb 28;11:82. doi: 10.3389/fpsyt.2020.00082 (PMC7058799; doi:10.3389/fpsyt.2020.00082)
Supplement: Supplementary file 6 [file Table_3.doc]

**Table S**3. Linear mixed model results for the subjective sleep questionnaires

|  | **Interaction effect** | **Main effects** |  | **Significant post-hoc effects^A^** |
| --- | --- | --- | --- | --- |
| **Variable** | **Time * Treatment** | **Treatment** | **Time** |  |
| ELIT mood | F(2,91)=0.08 P=0.923 | F(1,51)=0.19  P=0.663 | F(2,90)=2.14  P=.123 |  |
| ELIT sleep | F(2,93)=1.01  P=0.369 | F(1,54)=0.471  P=0.496 | F(2,93)=7.54  ***P=0.001*** | BL > DT > PT |
| ELIT wake | F(2,92)=2.04  P=0.135 | F(1,53)=0.07  P=0.797 | F(2,92)=4.07  ***P=0.020*** | BL > PT |
| ESS | F(2,87)=0.08  P=0.920 | F(1,49)=5.21  ***P=0.027*** | F(2,87)=1.57  P=0.214 | PLAC > MPH |

Abbreviations: BL, baseline; DT, during treatment; MPH, methylphenidate; PLAC, placebo; PT, post-treatment

^A^ Sidak post-hoc test P<0.05
